# Summary of the Course of Demonstrative Surgery in the University of Pennsylvania, from December 14, 1850, to January 18, 1851

**Published:** 1851-02

**Authors:** 


### CLINICAL REPORTS.

---

*Summary of the Course of Demonstrative Surgery in the University of Pennsylvania, from December* 14, 1850, *to January* 18, 1851. (Reported for the "Examiner.")

In continuing our Report of the cases and operations presented to the Medical Class of this Institution, (see No. 1, Jan. 1851, of this Journal,) the general arrangements of the course should be borne in mind. Its object is to increase the experience and practical knowledge of the student, by presenting him with cases of marked interest, either in diagnosis, pathology, or practice. No attempt is therefore made to show a number of patients, experience having proved that one or two operations, together with the remarks on the peculiarities of important cases, is more than sufficient to occupy sixty minutes, the period alloted each day to this portion of the course of Demonstrative instruction.

For the study and examination of the ordinary cases of surgery, an opportunity is afforded in the Dispensary of the Institution, where abscesses, ulcers, varicose veins, ophthalmiæ, &c., are sufficiently common. From the number of cases there collected, and from the private patients of the Lecturers, those shown to the class on Wednesday and Saturday, are selected, with a special view to their practical value. But as the readers of a journal may justly expect more than a detail of matters, which though highly useful to a student, could not possibly be so to a practitioner, such cases have been culled from those shown since the last report, as seemed most worthy of note, as well as illustrative of the value of this kind of instruction.

*Case of Molluscum Contagiosum—Amputation.*

In our last report, reference was made to an old lady who had labored under a disease believed to be Molluscum, and from whose arm two of the tumors were removed. Having now recovered fully from that operation, she was again brought forward by Dr. Smith, in consequence of a change in her symptoms, which required further treatment. The arm above the elbow was now perfectly sound; the axillary glands and lymphatics healthy: the patient's digestion, &c. good, but she had been much exhausted by hæmorrhage, consequent on sloughing in the ulcer below the elbow. She now showed no signs of disease elsewhere; but the whole fore-arm was so involved in the tumors, and in the sloughing ulcer at its upper third, that little or no hope of the recovery of the usefulness of the limb, was entertained. As the sloughing had also opened the veins at the bend of the elbow, and the ulcer extended around two-thirds of the fore-arm, Dr. Smith stated, that a consultation had decided that amputation furnished the best means of arresting the disease. Accordingly, after perfect etherisation (by the anæsthetic mentioned in our last report,) the Doctor removed the limb above the elbow, by the ordinary circular operation. During its performance, as well as throughout the dressing, the patient remained, as she subsequently stated to the class, unconscious of all that was done. No bad symptom followed, and the stump was sufficiently healed in fourteen days, to enable her to return to her home.

On an examination of some of the tumors, by the microscope, Dr. Joseph Leidy found that they presented numerous nucleated cells, with encephaloid matter so intermingled with fibres, &c., as to leave but little doubt of the malignant character of the disease.

*Fungus Hæmatodes of the Thigh.*

On the renewal of the course, after the vacation of Christmas, Dr. Horner presented a gentleman laboring under a fungous tumor on the outside of the thigh, just above the knee. This tumor had commenced in a very unsuspicious manner, about eighteen months since, but had lately rapidly increased and now presented well-marked characteristics of the medullary fungus of Müller. An operation was theretore left for further consideration.

*Calculus in the Bladder—Operation of Lithotripsy.*

Dr. Horner also presented (January 4th) a patient laboring under Stone in the Bladder, which had existed several months. After detailing the history of its formation, &c., the Doctor introduced Heurteloup's instrument, and immediately seized the stone, on the right side of the bas-fond of the bladder. After crushing it three times, he withdrew the instrument, the patient exhibiting so little inconvenience, as to be able to return to his home. In the previous soundings of this patient, little sensibility was shown, and it was, therefore, not thought necessary to etherise him. During the operation and up to this date, he has suffered no further trouble than that arising from the lodging of a fragment in the prostatic portion of the urethra, from which, however, it was readily removed and returned into the bladder.

At the same time, Dr. Horner removed several large nasal polypi, by means of the wire ligature of Physic, and double canula of Levret. These polypi were of the soft or moist kind—consisted of a moist, homogeneous tissue, covered by a thin process of mucous membrane, and contained in their cells a mucous or sero-mucous fluid, which, on being evacuated, left only the mucous, or skin-like covering.

Patients suffering from Carbuncle, Scirrhus of the Tonsils, Fracture of the Olecranon, Encysted Tumors, &c., were presented and treated between this and the next date, but offered nothing worthy of general notice.

*Amputation of the Thigh.*

On the 11th of January, Dr. Smith brought forward a patient laboring under a disease of the knee, which was believed to be scrofulous in its character. Two years since, this patient had fallen, whilst running, and struck his knee against the curb-stone. Violent inflammation ensued, accompanied by swelling around the joint, and followed by extensive suppuration in the ham. After some months, finding himself no better, he entered the Pennsylvania Hospital, where he remained five months, but left the institution uncured. He was then treated at his home, until April, 1850, when he entered the St. Joseph's Hospital, a new institution, in the northern portion of the city, under the care of the Sisters of Mercy. The leg was then bent nearly to a right angle with the thigh; a long, superficial ulcer existed in the ham, and extension of the limb could not be accomplished. His general health was also much impaired, and the discharge from the ham was disposed to burrow beneath the fascia. Under constitutional treatment and the use of Stromeyer's splint, the leg, however, was gradually extended: the ulcer healed with the exception of a small spot, and he was enabled to walk about, with the splint and the aid of his crutch. In July he left the Hospital and returned home; soon after which he had a return of swelling in the knee, for which he was advised to consult a great "knee-doctor," by whose direction the splint was removed, and stimulants applied around the joint. It is hardly necessary to say, that he soon got worse. The leg resumed the flexed position; suppuration again became free in the ham, and an abscess, believed to be exterior to the joint, opened in front, and on the side of the patella: hectic fever became developed, and being exhausted in body and mind, he determined to seek relief in amputation.

On a careful examination of the limb, the operation was deemed advisable, and after etherization, Dr. Smith, assisted by Dr. John Neill, removed the limb a few inches above the knee, by three circular sweeps of the knife, inclined obliquely upwards, so as form a hollow cone, of which the end of the bone was the apex; the first through the skin, which was forcibly retracted—the second through the first layer of muscles, also retracted—and the third down to the bone, as recommended by Alanson, and often practised in France in this amputation. On uniting the flaps, the stump appeared to be well formed and adapted to receive the artificial limb which the patient purposed to wear on his recovery. On dissection of the parts after the operation, the skin of the ham was found to be firmly adherent to the tissue beneath by cicatrices; the cellular structure indurated and infiltrated with lymph, cutting not unlike scirrhus; the tendons of the biceps, semi-membranosus and semi-tendinosus, were adherent to the surrounding parts, and the inflammation developed in the ham, had penetrated the joint from behind. The synovial capsule was dry and thickened, but without ulceration or disease over the cartilages, while the crucial ligaments and sides of the condyle at their origins, were so soft as to yield to the action of the forceps with great facility. The stiffness was therefore evidently the result of external inflammation, which had travelled into the joint from behind, and would probably have resulted in cartilaginous ulceration and bony anchylosis, if hectic had not previously destroyed the patient.

*Scirrhus of the Penis—Amputation.*

After this case was removed, Dr. Horner brought forward a patient suffering from Scirrhus of the Penis. From previous treatment in the Blockley Hospital, the progress of the disease had been from time to time arrested, though not more than one-fifth of the penis remained. The bulb and corpora cavernosa were now indurated; sloughing had destroyed the urethra nearly to this point, and the patient suffered constantly from pain, excoriation of urine, ulceration, &c. As the inguinal glands had never been affected, and his general condition required that some effort should be made to remedy the state of the parts, Dr. Horner determined to remove the crura of the penis and bulb ; separate the scrotum by a middle vertical incision, and thus form a canal from the pubis to the inferior extremity of the scrotum through the latter. Accordingly by a V shaped incision, the base downwards, he removed the remains of the organ; arrested the hæmorrhage, which was by no means severe, and thendividing the scrotum in front, at the raphè, formed a sort of vulva out of its two sides. The patient is now, (Jan. 20th,) doing well ; the ligatures have separated ; his urine has been passed through the wound, and the latter gives promise of speedy healing.

*Large Hydrocele, simulating Hernia.*

On the 18th Jan., Dr. Horner brought forward a man laboring under a large scrotal tumor, which extended up within the internal abdominal ring. About three inches below the external ring there was a partial contraction in the swelling, and then the tumor enlarged in a conical shape to the bottom of the scrotum. The patient stated that the complaint had existed two years ; first showed itself after a strain, and then gradually increased. It had never disappeared. Fluctuation was distinct throughout it ; there was no gurgling ; coughing created a marked motion in the whole tumor, even to its base, and a careful examination by the candle (transmitted light) showed it to consist of some dark or thickened substance. No light could be seen through the tumor at any point, and placing the patient on his back caused no change in its size. The diagnosis of the consultation was, therefore, hernial protrusion of intestine at the upper part of the tumor, and hydrocele with a thickened tunica vaginalis below. As there was however a possibility of the whole tumor being a hernia, it was decided to open it, layer by layer. This was accordingly done by Dr. Horner, by a careful dissection, until a thickened serous membrane was reached, lying just below a strongly developed cremaster muscle. On making a minute puncture, a small portion of fluid escaped, but it was quite sufficient to relieve the tension of the tumor and render the existence of hydrocele at this point certain. The sac therefore was punctured by a trocar and canula, and fully three pints of dark fluid drawn off, of about the color of a strong infusion of black tea. The whole tumor then disappeared. Thinking that the hernia might have been returned, the patient was placed in an erect position and directed to cough, but without causing any hernial protrusion. Finding something unusual, and fearing to inject the cavity if it opened or was connected with the belly, the Dr. introduced a long probe, through the canula, through the external ring and through the internal ring, two and a half inches, where its further passage was arrested, thus establishing the fact that the patient labored under a large hydrocele, which following the course of the cord through the inguinal canal, had been arrested by the reflection of the lining of the abdomen, and thus acted on, by the motion of the bowels, in coughing. Lest too violent an inflammation should be created by injecting so large a cavity, the parts were irritated by the pressure of a probe roughly moved around the tunica vaginalis, and the patient was directed to report himself as soon as the fluid collected again.

In connection with the peculiarities of this patient, Dr. Horner stated, that he had only once met with any thing analogous to the condition of parts just explained. This was in a gentleman who had visited the city several years since, in order to place himself under the care of Dr. Thos. Harris, U. S. Navy, now Chief of the Medical Bureau at Washington. This patient also had a large tumor in the scrotum about the size of the present one; but pressure from below readily diminished it one half, and the same effect was produced by placing him in the recumbent posture, the fluid passing up into the left iliac region. Being satisfied that it was a large hydrocele communicating directly with the interior of the pelvis, Dr. Harris punctured it, drew off about half a gallon of serum, and leaving a catheter in the cavity of the tunica vaginalis, induced inflammation without injecting it.

Patients suffering from a bursal tumor in the patella; from stricture of the œsophagus (boy æt. 10,) and from fistula in ano, were also shown and operated on, in the usual way. A singular case of deafness arising from a modification in the shape of the external meatus; a case of very bad hæmorrhoids with prolapsus, and some others, were also awaiting treatment at the close of the hour, and will be reported on a future occasion.

